# Notes on Preparations for the Sick

**Published:** 1909-03

**Authors:** 


### Notes on Preparations for the Sick.

**Calomel Cream; Roboleine; Pulverettes.**—OPPENHEIMER, SON & Co., London.—The **Calomel Cream** has been prepared at the suggestion of Colonel Lambkin, who has given it an extensive trial, and finds good results from its use by hypodermic intra-muscular injection. It is issued in aseptules containing the dose of fifteen minims, is absolutely sterile and causes no pain or local irritation. It contains five per cent. of Calomel.

**Roboleine** is a combination of fresh calf bone-marrow, with the expressed extract from ox ribs, and cream of malt with hypophosphates of lime, soda and potash. It is quite palatable, and its results are believed to be superior to those derived from cod liver oil and malt extracts.

**Pulverettes** have been designed as an improvement on the ordinary pill or tablet. They have a frail chocolate and sugar coating, but are easily crushed between thumb and finger. They are readily dissolved, and it is obviously impossible that they could pass through the alimentary canal and remain entire, as not unfrequently happens with coated pills, tablets and even gelatine capsules. A long list of drugs is now available for use in the form of Pulverettes. The makers claim that they are absolutely accurate in weight, and may be looked upon as a British pharmaceutical triumph.

---

**Iodival:** A monoiodisovalerianyl urea. **Digipuratum:** Extractum Digitalis depuratum.—KNOLL & CO., Harp Lane, E.C.—**Iodival** is an organic preparation containing 47 per cent. of iodine. This drug passes through the stomach unchanged; but in the intestine, in the presence of sodium hydrate, it enters into solution as a sodium salt. The liberation of iodine from this sodium salt proceeds gradually, so that it takes some days for all the sodium to be given off. The action of the iodine, therefore, lasts for several days, and will be of use in cases of syphilis, arterio-sclerosis, asthma, &c.

**Digipuratum** is a preparation of digitalis uniform in action and stable in composition, whose activity has been physiologically attested. It is put up as a powder corresponding in strength to powdered digitalis leaves, and also in tablet form, each one being equal to 0.1 grm. of digitalis leaves. As a rule, four tablets on the first day, three tablets on the second and third days, and two tablets on the fourth day, half an hour after meals, are sufficient. If a pronounced effect is secured on the second day, two tablets will suffice for the third day.

---

**Important Arylarsonates.**—BURROUGHS, WELLCOME & CO., London.—Striking clinical results are said to have been obtained with the new arylarsonates, known under the trade-mark names of "Soamin," "Kharsin" and "Orsudan."

In the treatment of syphilis, sleeping sickness, malaria and other protozoal diseases the low toxicity of these salts enables physicians to administer comparatively large quantities of arsenic without any toxic effects.

**"Soamin" (Sodium Para-aminophenylarsonate)** is stable, uniform in action, is soluble in about five parts of water, and gives a neutral solution which can be sterilised. It contains 22.8 per cent. of arsenium in organic combination, and has less than $\frac{1}{40}$th the toxicity of arsenious acid. Results of the administration of "Soamin" in cases of syphilis demonstrate the great therapeutic value of this agent.

**"Kharsin" (Sodium 3—methyl 4—aminophenylarsonate)** is soluble in two and a half times its weight of water, and gives a neutral solution. It contains 23.7 per cent. of arsenium, and is about equal in toxicity to "Soamin."

**"Orsudan" (Sodium 3—methyl 4—acetylaminophenylarsonate)** is soluble in three times its weight of water, and gives a neutral solution. It contains 25.4 per cent. of arsenium, and is the least toxic of the three salts. It has proved by recent clinical trials to be of marked value in malaria.

The arylarsonate salts should not be given by the mouth, as they are broken up by the acid contents of the stomach, and the effects of over-treatment by arsenic are thus more easily produced. Freshly prepared solutions should be administered by subcutaneous or intra-muscular injection, preferably the latter.

"Soamin" should not be used simultaneously with mercury, nor administered until fifteen days after mercurial treatment has ceased.

These drugs have yet to establish themselves. They appear to pass through the body unchanged, and it is doubtful whether their action is due in any way to the arsenic which they contain in so large a quantity.

---

**New "Tabloid" Hypodermic Preparations.**—BURROUGHS, WELLCOME & CO., London.—The following combinations are now issued :—

"Tabloid" Hypodermic, No. 121.

| | | |
|---|---|---|
| Atropine Sulphate .. | .. | gr. 1.200 |
| Strychnine Sulphate | .. | gr. 1.100 |

"Tabloid" Hypodermic, No. 122.

| | | |
|---|---|---|
| Atropine Sulphate .. | .. | gr. 1.150 |
| Strychnine Sulphate | .. | gr. 1.80 |

These preparations are of service in the treatment of inebriety and narcomania. The hypodermic administration of small doses of atropine and strychnine for a few weeks has been found to create a repulsion for alcohol which is more or less lasting in character.

---

**Elixir Lactopeptine.**—RICHARDS, 46 Holborn Viaduct, E.C.—Each fluid ounce represents :—

Lactopeptine, gr. 38, with alcohol 19 per cent.

One of the uses of this palatable combination is to disguise the disagreeable properties of other drugs such as sodium salicylate, chloral hydrate, antipyrin, ergot, the iodide and bromide salts, &c.

---

**Autan; Guaiacose.**—The Bayer Company, Elberfeld and London.—Autan is designed for the convenient application of formaldehyde to disinfection by fumigation. Practically no apparatus is required, there is no risk of fire, no difficulty of storage or transport, skilled labour is unnecessary, and many simultaneous disinfections can be carried out.

The method consists in the mere addition of water to Autan as a means of evolving formaldehyde and steam. The original package and a pail are the only apparatus required. It has been recently awarded a medal by the Royal Sanitary Institute, and it is in use largely by corporations, shipping and railway companies, &c.

**Guaiacose** (Liquid Guaiacol Somatose).—This combination should be of great use in the treatment of respiratory diseases, more particularly pulmonary tuberculosis. It needs no commendation, as a combination of the well-known Somatose with Calcium-Guaiacol-Sulphonate (a tasteless and odourless form of Guaiacol), in liquid form with an agreeable flavour, cannot fail to be of use to the doctor and his patients.

---

**Tannismut.**—E. W. Blasius, London.—This chemical combination of bismuth with tannic acid is made by the Von Heyden Company, of Radebeul, Dresden. It is said to be the most efficacious remedy in all forms of diarrhœa: one molecule of tannic acid splits off more readily than the other, and hence the astringent action may extend along the entire length of the bowel. It is more agreeable to the taste than its name would imply. Why is it necessary to invent such ill-sounding and meaningless appellations? We have found it to be of some use in a case of prolonged mucous colitis, in which the ordinary preparations of bismuth and many other drugs have been of no effect.

